# Hepatocyte‐Targeted Lipid Nanoparticle Delivery of HERC2 Plasmid Controls Drug‐Induced Hepatotoxicity by Limiting β‐Catenin‐Regulated CYP2E1 Expression

**DOI:** 10.1002/advs.202401633

**Published:** 2024-10-23

**Authors:** Yunzhi Liu, Qishan Xu, Yan Liu, Sihang Cao, Jialiang Luo, Zhuojun Zheng, Jia Zhou, Xiao Lu, Liyun Zhang, Yanan Tan, Qingyun Chen, Daming Zuo

**Affiliations:** ^1^ Institute of Molecular Immunology School of Laboratory Medicine and Biotechnology Southern Medical University Guangzhou Guangdong 510515 China; ^2^ Clinical Oncology Center Shenzhen Key Laboratory for cancer metastasis and personalized therapy The University of Hong Kong‐Shenzhen Hospital Shenzhen Guangdong 518053 China; ^3^ Shenzhen Institute of Advanced Technology Chinese Academy of Sciences Shenzhen 518055 China; ^4^ Department of Precision Laboratory Affiliated Hospital of Guangdong Medical University Zhanjiang Guangdong 510180 China; ^5^ Department of Microbiology Li Ka Shing Faculty of Medicine The University of Hong Kong Pokfulam Hong Kong SAR 999077 China; ^6^ Guangdong Province Key Laboratory of Proteomics Department of Immunology School of Basic Medical Sciences Southern Medical University Guangzhou Guangdong 510515 China; ^7^ Medical Research Institute Guangdong Provincial People's Hospital Guangdong Academy of Medical Sciences Southern Medical University Guangzhou Guangdong 510080 China; ^8^ Guangdong Province Key Laboratory of Immune Regulation and Immunotherapy School of Laboratory Medicine and Biotechnology Southern Medical University Guangzhou Guangdong 510515 China; ^9^ Advanced Energy Science and Technology Guangdong Laboratory Huizhou Guangdong 516001 China

**Keywords:** CYP2E1, drug‐induced liver injury, HERC2, lipid nanoparticle, β‐catenin

## Abstract

Understanding the molecular mechanisms that bridge hepatic inflammation and liver injury is crucial for developing effective therapeutic strategies for drug‐induced liver injury (DILI) management. HECT domain and RCC1‐like domain 2 (HERC2) belongs to the large Herc family of ubiquitin E3 ligases, which are implicated in tissue development and inflammation. The observation reveals a pronounced HERC2 expression in specific hepatocyte subsets that proliferate in response to DILI in humans, prompting an investigation into the role of HERC2 in distinct DILI progression. Under the APAP challenge, liver‐specific HERC2‐deficient mice suffer more severe liver damage. Integrated single‐cell RNA sequencing analysis unveils a negative correlation between HERC2 and CYP2E1, a vital metabolic enzyme for xenobiotics, in hepatocytes from APAP‐challenged mice. Mechanistically, HERC2 interacts with β‐catenin to promote its ubiquitination, thereby governing CYP2E1 transcriptional regulation. Targeted hepatic delivery of lipid nanoparticle‐encapsulated HERC2‐overexpressing plasmid markedly reduces liver damage caused by APAP overdose. Collectively, these findings elucidate a previously unrecognized protective role of HERC2 in protecting against acute liver injury associated with drug metabolism disorders, highlighting its potential as a therapeutic target in treating DILI.

## Graphical abstract

The investigation revealed pronounced HERC2 expression in specific hepatocyte subsets that proliferate in response to APAP‐induced liver injury in human. It is demonstrated that HERC2 protected hepatocytes from APAP‐induced hepatoxicity in mice. Mechanistically, HERC2 interacts with β‐catenin to promote its ubiquitination, thereby governing CYP2E1 transcription. Notably, targeted hepatic delivery of lipid nanoparticle‐encapsulated HERC2‐overexpressing plasmid markedly reduced liver damage caused by APAP overdose.

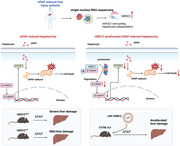

## 1 Background

The widespread utilization of pharmaceuticals, coupled with the ongoing introduction of novel medications, has elevated concerns regarding drug safety. Drug‐induced liver injury (DILI) is one of the most prevalent and severe adverse drug reactions, which can be caused by medications, herbal products, or dietary supplements, ranging from mild liver enzyme elevations to liver failure.^[^ ^1^ ^]^ To date, there is still a deficiency of indexes for straightforward, objective, and specific diagnosis, as well as specific therapeutic approaches for DILI.^[^ ^2^ ^]^ The pharmacotherapy of DILI presents significant challenges due to its complex pathophysiology and the variability of patient responses. Given the critical role of the liver in drug metabolism, DILI management requires a nuanced approach that balances the need for continued medication with the potential for hepatic recovery.^[^ ^1^, ^2^ ^]^ Notably, various distinct mechanisms result in hepatotoxicity, including cell membrane disruption and cell death resulting from covalent binding of the drug to cell proteins, creating new adducts that serve as immune targets to elicit an inflammatory reaction.^[^ ^1^, ^3^ ^]^ Acetaminophen (APAP) is one of the most commonly consumed over‐the‐counter drugs but is also a significant cause of acute liver injury.^[^ ^1^ ^]^ APAP is metabolized predominantly by sulfation and glucuronidation to inactive conjugates. *N*‐Acetyl‐*p*‐benzoquinone imine (NAPQI) is a reactive metabolite of APAP formed primarily by the cytochrome P450 (CYP) metabolic pathway.^[^ ^4^ ^]^ NAPQI binds to mitochondrial proteins, resulting in mitochondrial dysfunction, reactive oxygen species (ROS) overproduction, and cell death.^[^ ^5^ ^]^ N‐acetylcysteine (NAC) is currently the only FDA‐approved therapeutic option for APAP overdose, while it has notable limitations, including adverse effects and a narrow therapeutic window.^[^ ^6^ ^]^ As a result, the search for novel therapeutic targets in DILI continues to be necessary.

CYP2E1 is a drug‐metabolizing enzyme, exclusively expressed in hepatocytes surrounding branches of the hepatic central vein, which plays a vital role in drug‐induced hepatotoxicity.^[^ ^4^, ^7^ ^]^ The *CYP2E1* gene is inducible in exposure to active compounds such as ethanol, isoniazid, and other compounds.^[^ ^8^ ^]^ Upregulated CYP2E1 has multiple pathophysiological roles in the liver, including increased oxidative stress‐mediated apoptosis and cell death, as well as energy supply to meet the energy demand of the liver in certain disease states.^[^ ^9^ ^]^ Notably, transcription factors, nuclear receptors (NRs), co‐activators, and corepressors are critical transcriptional regulators of *CYP* expression. For instance, the transcription factor hepatocyte nuclear factor 1 alpha (HNF‐1α) can bind to the promoter region of CYP2E1 and enhance its transcription.^[^ ^7^ ^]^ Moreover, HNF‐4α also binds to the promoter region of CYP2E1 and affects CYP2E1 expression levels.^[^ ^10^ ^]^ Gerbal‐Chaloin et al., observed that the WNT/β‐catenin pathway transcriptionally regulated CYP2E1 expression, and the expression of CYP2E1 was almost completely abolished in the livers of hepatocyte‐specific β‐catenin knockout mice.^[^ ^11^ ^]^

HECT and RLD domain‐containing E3 ubiquitin protein ligase 2 (HERC2) belongs to the large HERC family of ubiquitin E3 ligases with multiple structural domains implicated in a wide range of physiological processes, including inflammation, immune response, DNA repair, and cell stress response.^[^ ^12^ ^]^ The HERC protein family regulates ubiquitination and interferon‐stimulated gene 15 conjugation (ISGylation) processes associated with inflammatory responses and cancer biology.^[^ ^12^, ^13^ ^]^ It has been reported that HERC2 shuttled between the nucleus and cytoplasm and functions as an E3 ubiquitin ligase for the ubiquitination and degradation of target proteins. Wu et al. showed that HERC2 ubiquitinates the tumor suppressor breast cancer susceptibility gene 1 (BRCA1) in relation to the cell cycle and DNA damage response.^[^ ^14^ ^]^ HERC2 also promotes the degradation of the liver kinase B1 (LKB1) when it is acetylated.^[^ ^15^ ^]^ Our previous study demonstrated that inflammatory stimulation promoted HERC2 expression in hepatocytes and that elevated HERC2 expression was associated with the progression and poor prognosis of hepatocellular carcinoma (HCC).^[^ ^12a^ ^]^ However, the role of HERC2 in the progression of DILI and the regulation of drug‐induced hepatotoxicity remains unclear.

In this study, using liver‐specific HERC2‐deficient (HERC2*^∆Alb^*) mice, we found that abrogation of HERC2 exacerbated APAP overdose‐induced liver injury through increasing inflammatory responses, ROS production, and hepatocyte death. HERC2 deficiency promoted the expression of CYP2E1 in hepatocytes upon drug challenge in both in vivo and in vitro analyses. We further identified that HERC2 interacted with β‐catenin and promoted its ubiquitination, consequently transcriptionally regulating the expression of CYP2E1. Importantly, we demonstrated that targeted delivery of lipid nanoparticle (LNP)‐encapsulated HERC2‐overexpressed plasmids into hepatocytes significantly attenuated the APAP overdose‐induced liver damage, indicating that HERC2 could be a new therapeutic target of DILI.

## 2 Results

### 2.1 Hepatocyte HERC2 Deficiency Exacerbated APAP‐Induced Acute Liver Injury

**Figure 1:**
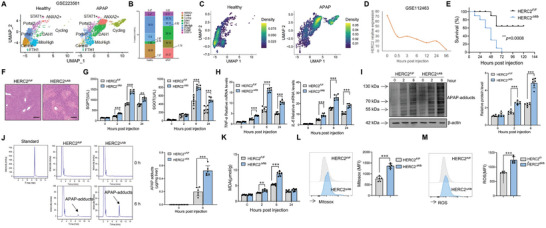
Hepatocyte HERC2 deficiency exacerbated APAP‐induced liver damage in mice. A–C) A single‐cell dataset (GSE223581) was used to identify HERC2 expression patterns in patients with DILI. A) Dimplot indicated hepatocyte clusters. B) Different cluster ratio was displayed. C) DensityPlot showed HERC2 expression in hepatocytes from healthy controls and patients with DILI. D) Genome‐wide CRISPR‐Cas9 Screen (GSE112463) data analysis indicated the role of HERC2 in the progression of APAP‐induced acute liver injury. E) HERC2*^F/F^* and HERC2*^∆Alb^* mice were starved for 16 hours, and intraperitoneally injected with APAP at a dose of 700 mg kg^−1^, and the overall survival of the mice was monitored (n = 10). F–L) APAP (400 mg kg^−1^) was injected into HERC2*^F/F^* and HERC2*^∆Alb^* mice (n = 6). F) Histological analysis of mouse livers was performed with H&E staining at 24 hours upon APAP challenge, scale bars = 100 µm. G) The serum SGPT and SGOT activities were measured at different time points after APAP administration. H) The mRNA expression levels of TNF‐α and IL‐6 in liver tissues were determined by quantitative RT‐PCR assay. I) APAP adducts in the liver were evaluated by western blotting at the indicated time point post‐APAP injection. J) APAP‐adducts level was determined by HPLC assay from liver tissues at 6 hours post APAP treatment. K) MDA level in liver tissues was detected. Flow cytometry analysis of mitochondrial ROS using MitoSOX (L) and intercellular ROS level by the fluorescent probe DCFH‐DA (M) in hepatocytes was carried out at the indicated time points after APAP injection. **p < 0.01, ***p < 0.001. Data from one representative experiment of three independent experiments are presented. The data are displayed as the mean ± standard deviation (SD). Kaplan–Meier and log‐rank tests and unpaired Student's *t*‐test were used.

Hepatocytes, the central parenchymal cells in the liver, are responsible for maintaining liver function. We first investigated the expression of HERC2 in human liver explant samples from patients transplanted for APAP‐induced acute liver failure with an opened snRNA‐seq database (GSE223581). The data revealed that the clustering of the ANXA2^+^ subpopulation and cycling hepatocyte subpopulation was presented in patients with APAP‐induced acute liver failure compared to healthy controls (**Figure** **1**A,B). HERC2 expression was mapped by the density plot. Surprisingly, HERC2 expression was highly concentrated in both ANXA2^+^ hepatocytes and cycling hepatocytes during APAP hepatoxicity (Figure 1C). In recent years, the unbiased functional genomic Crispr‐Cas9 screening method has rapidly revolutionized the field of functional genomics.^[^ ^16^ ^]^ In a Crispr‐Cas9 knockout library, a positive screen identified these genes potentially increased susceptibility to the treatment, while a negative screen suggested the genes were essential to survival of the treatment condition. Notably, data from a public genome‐wide Crispr‐Cas9 screen (GSE112463) analysis indicated that HERC2 was identified as a negative screen, suggesting a protective role of HERC2 in APAP‐induced liver injury (Figure 1D). To investigate the precise role of HERC2 in APAP‐induced liver injury, HERC2*^F/F^* and HERC2*^∆Alb^* mice were given a lethal dose of APAP (700 mg kg^−1^) by intraperitoneal injection. The survival analysis revealed that HERC2*^F/F^* mice displayed a significant survival advantage compared to HERC2*^∆Alb^* mice (Figure 1E). Next, HERC2*^F/F^* and HERC2*^∆Alb^* mice were intraperitoneally injected with APAP at a dose of 400 mg kg^−1^. H&E staining showed that the HERC2*^∆Alb^* groups exhibited apparent destruction of hepatic lobule structure compared to HERC2*^F/F^* groups upon APAP challenge (Figure 1F). Furthermore, the levels of SGPT and SGOT in serum were higher in APAP‐treated HERC2*^∆Alb^* mice compared to APAP‐treated HERC2*^F/F^* mice (Figure 1G). Lack of HERC2 in hepatocytes markedly elevated intrahepatic levels of pro‐inflammatory cytokines (e.g., IL‐6 and TNF‐α) after the APAP administration (Figure 1H). Also, the expression of APAP protein adduct was significantly increased in the liver of HERC2*^∆Alb^* mice compared to control mice after APAP injection as indicated by the western blot and HPLC analysis (Figure 1I,J). Toxic metabolites of APAP exacerbated oxidative stress in the liver, and the production of mitochondrial ROS triggered by APAP is a critical event in APAP hepatotoxicity.^[^ ^5^, ^17^ ^]^ Thus, we further explore whether HERC2 affected the level of oxidative stress in the liver after APAP stimulation. Liver tissues from APAP‐treated HERC2*^∆Alb^* mice at different time points showed significantly increased levels of malondialdehyde (MDA) compared with those from control mice (Figure 1K). Additionally, we observed more ROS production in the mitochondria from hepatocytes of HERC2*^∆Alb^* mice than in those of HERC2*^F/F^* mice with APAP administration (Figure 1L). We also tested ROS expression in liver cells and found elevated ROS levels in APAP‐treated HERC2*^∆Alb^* mice compared to their counterparts (Figure 1M). These results indicated a potential protective role of HERC2 in the pathogenesis of APAP‐induced liver damage.

**Figure 2:**
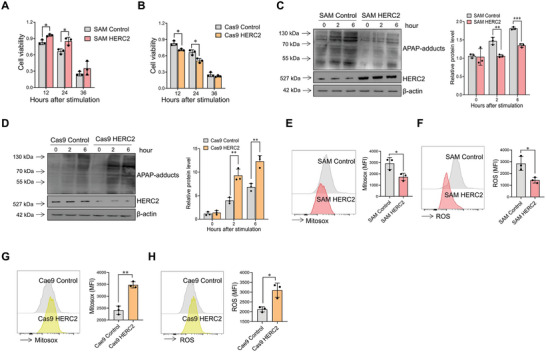
HERC2 protected against APAP‐induced cell injury in HepaRG cells. HERC2 overexpressed (SAM HERC2) or deficient (Cas9 HERC2) HepaRG cells were treated with APAP at doses of 20 mm. A,B) The cell viability was determined by the CCK‐8 assay. C,D) The levels of APAP‐adducts were evaluated by western blotting. E,G) Mitochondria ROS level was detected by flow cytometry assay. F,H) Intracellular ROS level was detected by flow cytometry assay. *p < 0.05, **p < 0.01, ***p < 0.001. Data from one representative experiment of three independent experiments are presented. The data are displayed as the mean ± standard deviation (SD). Unpaired Student's *t*‐test was used.

We then tried to clarify the effect of HERC2 on APAP‐induced acute liver injury in vitro. HERC2 overexpressing and HERC2 knockout HepaRG cells were established by the CRISPR‐Cas9 system. As indicated by CCK‐8 assays, overexpression of HERC2 increased HepaRG cell viability (**Figure** **2**A), whereas knockout of HERC2 significantly declined the cell viability of HepaRG cells (Figure 2B). Immunoblotting assay demonstrated that overexpression of HERC2 in HepaRG cells inhibited the expression of APAP protein adducts (Figure 2C). In contrast, HERC2 deficiency boosted APAP protein adducts expression (Figure 2D). HERC2‐overexpressed cells exhibited attenuated mitochondrial ROS production compared to the control cells upon APAP stimulation (Figure 2E). In addition, the ROS production in APAP‐stimulated HepaRG cells was significantly attenuated when HERC2 was overexpressed (Figure 2F). By contrast, HERC2 deficiency promoted the oxidative responses in hepatocytes upon APAP stimulation, as indicated by enhanced mitochondrial and cytoplasmic ROS production (Figure 2G,H). The observations in vitro also indicated that HERC2 could protect hepatocytes from oxidative stress caused by APAP administration.

### 2.2 HERC2 Regulated CYP2E1 Expression in Hepatocytes with APAP Toxicity

**Figure 3:**
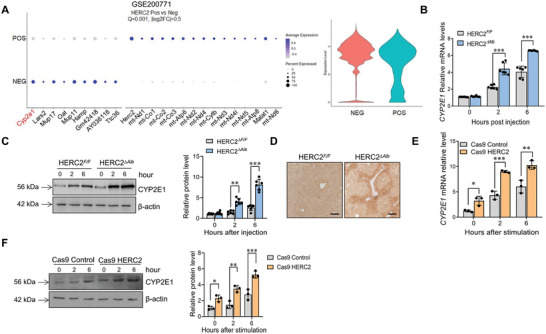
HERC2 limited CYP2E1 expression in hepatocytes. A) Public single‐cell sequencing data (GSE200771) was used to assess the major differential genes between HERC2 positive and negative hepatocytes. B–D) HERC2*^F/F^* and HERC2*^∆Alb^* mice were intraperitoneally injected with APAP (400 mg kg^−1^) (n = 6). B) The mRNA level of CYP2E1 in the liver tissues was detected by quantitative RT‐PCR analysis. The protein level of CYP2E1 in the liver tissues was evaluated by western blotting (C) and immunohistochemical staining (D), scale bars = 100 µm. The slides were originated from mice at 6 hours post APAP treatment. E,F) HERC2‐deficient (Cas9 HERC2) HepaRG cells and control cells were treated with APAP at the dose of 20 mM. E) The mRNA level of CYP2E1 was detected by quantitative RT‐PCR analysis. F) The protein level of CYP2E1 was evaluated by western blotting. *p < 0.05, **p < 0.01, ***p < 0.001. Data from one representative experiment of three independent experiments are presented. The data are displayed as the mean ± standard deviation (SD). Unpaired Student's *t*‐test was used.

Next, public single‐cell sequencing data (GSE200771) was used to assess the significant differential genes between HERC2‐positive and negative hepatocytes. We found that the HERC2‐positive group expressed a reduced level of CYP2E1 expression compared to the HERC2‐negative group (**Figure** **3**A). RT‐PCR results validated that the mRNA levels of CYP2E1 were higher in the liver of HERC2*^∆Alb^* mice than HERC2*^F/F^* mice after APAP challenge (Figure 3B). Moreover, immunoblotting results showed that HERC2*^∆Alb^* mice had higher protein expression levels of CYP2E1 than HERC2*^F/F^* mice (Figure 3C). Compared to HERC2*^F/F^* mice, APAP‐injected HERC2*^∆Alb^* mice had increased hepatic CYP2E1 protein levels, according to the results of immunohistochemistry staining (Figure 3D). We also evaluated the regulation of CYP2E1 by HERC2 with in vitro analysis. As shown, higher mRNA and protein levels of CYP2E1 were observed in HERC2‐deficient cells than control cells after APAP stimulation (Figure 3E,F). In contrast, HERC2 overexpression significantly reduced both mRNA and protein levels of CYP2E1 in hepatocytes following APAP administration (Figure S1A,B, Supporting Information). Cumulatively, these results suggested that HERC2 reduced the hepatic expression of CYP2E1 upon APAP challenge.

### 2.3 HERC2 Affected the Protein Expression of β‐Catenin, a Novel Transcription Factor of CYP2E1

**Figure 4:**
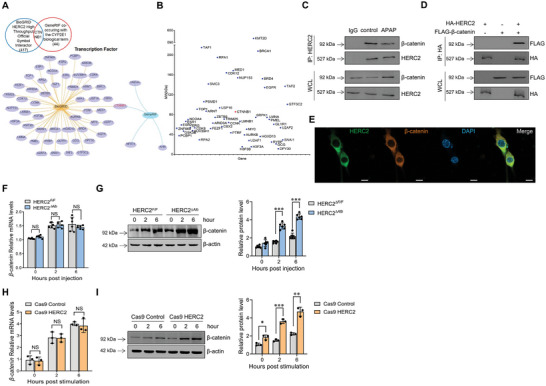
HERC2 interacted with β‐catenin and regulated β‐catenin protein level. A,B) Venn diagram (A) and dotplot (B) displayed the overlap between the HERC2‐interacted protein obtained from BioGRID database and CYP2E1 co‐occurred protein in the biological term acquired from GeneRIF. C) HepaRG cells were treated with 20 mm APAP for 6 hours, and an immunoprecipitation assay validated the interaction between HERC2 and β‐catenin. D) HEK293T cells were co‐transfected with HERC2‐HA and β‐catenin‐FLAG plasmids. Immunoprecipitation was performed using anti‐HA magnetic beads. The presence of coprecipitated β‐catenin was determined by immunoblotting with the anti‐FLAG antibody. E) The co‐localization of HERC2 and β‐catenin was analyzed by immunofluorescence assay, scale bars = 10 µm. F,G) HERC2^F/F^ and HERC2^∆Alb^ mice were intraperitoneally injected with APAP (400mg kg^−1^). F) The mRNA level of β‐catenin in the liver tissues was detected by quantitative RT‐PCR analysis. G) The protein level of β‐catenin in the liver tissues was evaluated by western blotting. H,I) HERC2‐deficient (Cas9 HERC2) HepaRG cells and control cells were treated with APAP at the dose of 20 mM. H) The mRNA level of β‐catenin in the cells was detected by quantitative RT‐PCR analysis. I) The protein level of β‐catenin in the cells was evaluated by western blotting. NS: not significant. *p < 0.05, **p < 0.01, ***p < 0.001. Data from one representative experiment of three independent experiments are presented. The data are displayed as the mean ± standard deviation (SD). Unpaired Student's *t*‐test was used.

As known, CYP2E1 expression is regulated by distinct transcription factors.^[^ ^7^, ^18^ ^]^ We, thus, analyzed the overlap between the HERC2‐interacted protein obtained from BioGRID database and the CYP2E1 co‐occurred protein in the biological term acquired from GeneRIF. Interestingly, β‐catenin (CTNNB1) was the only overlapping transcription factor found (**Figure** **4**A,B). We then confirmed the interaction between HERC2 and β‐catenin in HepaRG cells. Immunoprecipitation analysis displayed the binding of HERC2 with β‐catenin, and APAP stimulation reduced the interaction between HERC2 and β‐catenin (Figure 4C). To further identify the direct interaction between HERC2 and β‐catenin, HERC2 and β‐catenin plasmids were co‐transfected into HEK293T cells. Immunoprecipitation assays revealed a direct interaction between HERC2 and β‐catenin (Figure 4D). Moreover, immunofluorescence analysis further validated the direct binding of HERC2 to β‐catenin (Figure 4E). We subsequently investigated whether HERC2 regulates the expression of CYP2E1 via modulating β‐catenin activity. RT‐PCR results showed no statistical difference in β‐catenin mRNA levels between HERC2*^∆Alb^* and HERC2*^F/F^* mice (Figure 4F). Surprisingly, the protein expression levels of β‐catenin were higher in the liver tissues of HERC2^∆Alb^ mice compared to HERC2^F/F^ mice (Figure 4G). Next, in vitro experiments were performed to validate the regulatory effect of HERC2 on β‐catenin expression. Interestingly, no statistically significant differences in β‐catenin mRNA levels were found in HERC2 knockdown cells compared to control cells after APAP treatment (Figure 4H). However, higher protein levels of β‐catenin were found in APAP‐stimulated HERC2‐knockdown cells (Figure 4I).

**Figure 5:**
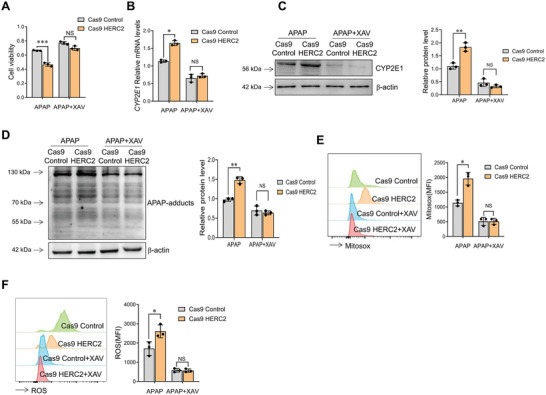
HERC2 interacted β‐catenin regulated APAP‐induced hepatoxicity. HERC2‐deficient (Cas9 HERC2) HepaRG cells and control cells were treated with 2 µM XAV939 for 4 hours before 20 mM APAP stimulation. A) The cell viability was determined by the CCK‐8 assay at 24 hours post APAP treatment. B‐F) The cells were harvested at 6 hours post APAP treatment. B) The mRNA level of CYP2E1 was detected by quantitative RT‐PCR analysis. C) The protein level of CYP2E1 and D) APAP‐adducts were evaluated by western blotting. E) The mitochondrial ROS level and F) intercellular ROS level in hepatocytes were determined by flow cytometry analysis. NS: not significant, *p < 0.05, **p < 0.01, ***p < 0.001. Data from one representative experiment of three independent experiments are presented. The data are displayed as the mean ± standard deviation (SD). Unpaired Student's *t*‐test was used.

To further explore whether HERC2 regulated CYP2E1 expression by affecting protein expression of β‐catenin, HepaRG cells were pre‐treated with XAV939, a β‐catenin inhibitor. According to CCK‐8 assay data, XAV939 significantly abolished the effect of HERC2 deficiency on cellular viability. The treatment elevated the cell viability of both APAP‐stimulated HERC2‐deficient and control cells (**Figure** **5**A). In addition, the effect of HERC2 on the mRNA and protein expression of CYP2E1 was abolished when cells were treated with XAV939 (Figure 5B,C). Comparable APAP‐adduct level was also observed when cells were treated with XAV939 (Figure 5D). Moreover, the expression level of mitochondrial ROS was downregulated once XAV939 was added, and the mitochondrial ROS activity was similar between APAP‐stimulated control cells and HERC2‐deficient cells when β‐catenin was blocked (Figure 5E). Additionally, regulation of ROS levels in hepatocytes by HERC2 was lost in the presence of XAV939 (Figure 5F). Moreover, XAV939 treatment abrogated the HERC2‐mediated regulation of CYP2E1 expression and subsequent oxidative responses, which is determined in the HERC2‐overexpressed cells (Figure S2, Supporting Information). Thus, we concluded that HERC2 alleviates APAP‐induced acute liver injury by reducing the expression of β‐catenin protein and consequently inhibiting the transcription of CYP2E1 in the liver upon APAP challenge.

### 2.4 HERC2 Facilitated the Degradation of β‐Catenin by the Proteasome

**Figure 6:**
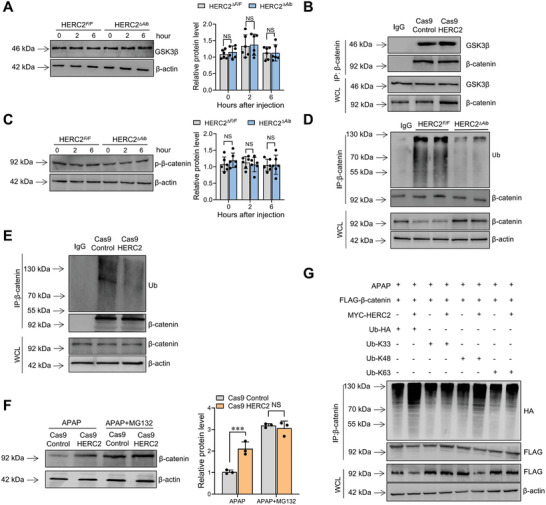
HERC2 promoted k48 ubiquitination of β‐catenin. A) HERC2*^F/F^* and HERC2*^∆Alb^* mice was intraperitoneally injected with APAP (400mg kg^−1^). The protein level of GSK3β was detected by western blot analysis. B) HERC2‐deficient (Cas9 HERC2) cells were treated with 20 mm APAP for 6 hours. An immunoprecipitation assay was performed to analyze the interaction between β‐catenin and GSK3β. C,D) HERC2*^F/F^* and HERC2*^∆Alb^* mice were intraperitoneally injected with APAP (400 mg kg^−1^). C) The phosphorylation level of β‐catenin in the liver tissues was determined by western blot analysis. D) The interaction between β‐catenin and Ub was validated by an immunoprecipitation assay. E,F) HERC2‐deficient HepaRG cells and control cells were treated with 20 mM APAP and 10 µM MG132 for 6 hours. E) The ubiquitination level of β‐catenin was determined by immunoprecipitation. F) The protein level of β‐catenin was evaluated by western blotting analysis. G) HEK293T cells were transfected with FLAG‐β‐catenin and MYC‐HERC2 combined with HA‐ubiquitin (Ub‐HA), HA‐ubiquitin‐K48, HA‐ubiquitin‐K63, or HA‐ubiquitin‐K33 plasmids for 48 hours. Then, cells were treated with 20 mm APAP and 10 µM MG132 for another 6 hours. The ubiquitination of β‐catenin was determined by immunoprecipitation. NS: not significant, ***p < 0.001. Data from one representative experiment of three independent experiments are presented.

The protein expression level of β‐catenin is mainly regulated by the β‐catenin degradation complex consisting of GSK3β. The β‐catenin degradation complex promotes the phosphorylation of β‐catenin and the subsequent ubiquitination degradation. We then tried to explore the effect of HERC2 on this pathway. No significant difference in protein expression of GSK3β was observed in the liver between APAP‐treated HERC2*^F/F^* mice and HERC2*^∆Alb^* mice (**Figure** **6**A). Immunoprecipitation assay showed that HERC2 did not affect the interaction between β‐catenin and GSK3β (Figure 6B). Moreover, phosphorylation levels of β‐catenin were comparable between the livers isolated from HERC2*^F/F^* mice and HERC2*^∆Alb^* mice upon APAP challenge (Figure 6C). These data indicated that HERC2 did not influence the GSK‐3β/β‐catenin signaling pathway. In addition, HERC2 also acts as an E3 ubiquitin ligase involved in the ubiquitination degradation process of the protein.^[^ ^12c^ ^]^ We, thus, tried to explore whether HERC2 affects the level of ubiquitination of β‐catenin. Immunoprecipitation assay showed that HERC2*^∆Alb^* mice had fewer ubiquitin molecules bound to β‐catenin compared to HERC2*^F/F^* mice (Figure 6D). Meanwhile, β‐catenin bound ubiquitin molecules were reduced in HERC2 knockdown cells (Figure 6E). In contrast, β‐catenin bound ubiquitin molecules were increased in HERC2 overexpressing cells (Figure S3A, Supporting Information). Next, we employed MG132, an inhibitor of the proteasome, to test whether HERC2 promotes β‐catenin degradation by the proteasome. The experimental results showed that the effect of HERC2 on β‐catenin expression abrogated after the addition of proteasome inhibitors (Figure 6F; Figure S3B, Supporting Information). Our results also demonstrated that HERC2 promoted polyubiquitination of K48‐linked in β‐catenin, leading to protein degradation (Figure 6G). Together, the results suggested that HERC2 promotes the degradation of β‐catenin by the proteasome through k48‐linked ubiquitination.

### 2.5 Targeted Delivery of LNP‐Encapsulated HERC2 Overexpressed Plasmids into Hepatocytes Attenuated Drug‐Induced Liver Injury

**Figure 7:**
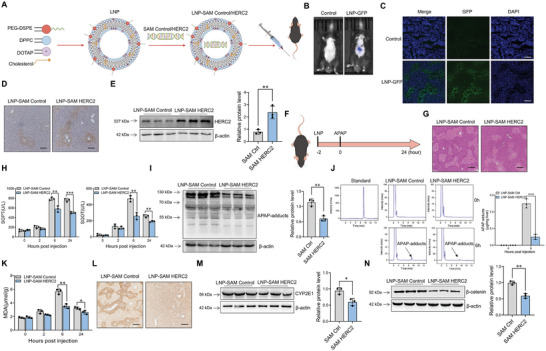
Targeted delivery of LNP‐encapsulated HERC2‐overexpressed plasmids into hepatocytes attenuated DILI in mice. A) Schematic diagram indicated the construction of plasmids‐loaded LNPs. B,C) 200µl LNPs were mixed with 20µg GFP labeled vector plasmids. The LNP‐DNA complex was injected into mice via tail vein for 24 hours. B) Animal live imaging showed the distribution of LNPs in vivo. C) The distribution of LNPs in the liver was detected by the fluorescence microscope, scale bars = 100 µm. D,E) 200µl LNPs were mixed with 20µg HERC2 overexpressed plasmids (SAM HERC2) or control plasmids (SAM Control). The LNP‐DNA complex was injected into mice via the tail vein. D) Immunohistochemical staining analysis indicated the expression pattern of HERC2 in the liver, scale bars = 100 µm. E) HERC2 expression in liver tissues was determined by western blotting analysis. F–N) The mice were injected with LNP‐encapsulated HERC2‐overpressed plasmid or control plasmid via tail vein. 2 hours later, the mice were intraperitoneally injected with APAP (400 mg kg^−1^) (n = 3) (F). G) Histological analysis of mouse livers at 24 hours post APAP treatment was detected with H&E staining, scale bars = 100 µm. H) The serum SGPT and SGOT level were measured. APAP‐adduct level in liver tissues at 6 hours post APAP treatment was detected by I) western blotting analysis and J) HPLC assay. K) MDA level in liver tissues was determined. The protein level of CYP2E1 in liver tissues at 6 hours post APAP treatment was evaluated by L) immunohistochemical staining and M) western blotting assay, scale bars = 100 µm. N) The protein level of β‐catenin in the liver tissues at 6 hours post APAP treatment was evaluated by western blotting. *p < 0.05, **p < 0.01, ***p < 0.001. Data from one representative experiment of three independent experiments are presented. The data are displayed as the mean ± standard deviation (SD). Unpaired Student's *t*‐test was used.

The lipid nanoparticle (LNP) delivery system is a revolutionary discovery that has enabled gene therapies and efficient mRNA vaccines.^[^ ^19^ ^]^ LNP has been considered for application in the treatment of non‐cancer liver diseases such as liver fibrosis.^[^ ^20^ ^]^ To test our hypothesis of whether HERC2 overexpressed plasmids delivered by LNP are able to mitigate DILI, we constructed LNP‐wrapped HERC2 overexpressed plasmids (**Figure** **7**A; Figure S4, Supporting Information). Plasmids encapsulated LNPs were mainly distributed in liver at 24 hours post injection (Figure 7B) and were dispersed near vein area (Figure 7C). The results showed plasmids encapsulated LNPs successfully promoted HERC2 expression in hepatocytes. (Figure 7D,E) We next investigated the regulatory function of HERC2‐overexpressed plasmid encapsulated LNPs in APAP hepatoxicity (Figure 7F). The pathology analysis indicated ameliorated liver damage in APAP‐injected mice treated with HERC2‐overexpressed plasmid encapsulated LNPs compared to the controls (Figure 7G). Consistently, declined serum SGPT and SGOT level was found in HERC2‐overexpressed plasmid encapsulated LNPs‐treated mice compared to control mice (Figure 7H). We also observed attenuated accumulation of APAP‐adducts (Figure 7I,J) and MDA (Figure 7K) in liver tissues when mice were treated with HERC2‐loaded LNPs . Moreover, HERC2‐loaded LNPs administration markedly decreased CYP2E1 expression in hepatocytes (Figure 7L,M), which was caused by impaired β‐catenin level (Figure 7N). Together, the data indicated that targeted delivery of HERC2‐overexpressed plasmid encapsulated LNPs attenuated DILI by modulating β‐catenin activity and consequent CYP2E1 expression.

## 3 Discussion

DILI represents a significant medical concern due to its association with hepatic impairment caused by certain pharmaceutical agents. This condition can trigger inflammation in the liver, which, if persistent, has the potential to escalate into liver dysfunction.^[^ ^1b^ ^]^ Thus, there is an urgent need to unravel molecular targets for hepatic inflammation that have potential in DILI therapy. Our previous study has elucidated the role of HERC2 in fostering inflammation‐driven cellular plasticity and immune evasion in HCC.^[^ ^12a^ ^]^ The current research newly demonstrated that elevated levels of HERC2 in hepatic tissue are essential in conferring protection against DILI. We observed a significant upregulation of HERC2 expression in the specific hepatocyte clusters expanded in APAP‐induced liver failure. Moreover, HERC2 exerts a regulatory effect on drug metabolism through the attenuation of β‐catenin degradation, which in turn diminishes CYP2E1 expression, thus mitigating subsequent drug‐induced hepatotoxicity.

HERC2 is a multifunctional protein involved in multiple cellular processes, including protein degradation, DNA damage response, and cell cycle regulation.^[^ ^12a,c^ ^]^ Our research has revealed that the absence of HERC2 in hepatocytes significantly increases the vulnerability of mice to DILI. This heightened susceptibility is underscored by a pronounced increase in mortality rates and the aggravation of liver pathology. The hypersensitivity to DILI observed in the absence of HERC2 correlates with an overproduction of pro‐inflammatory cytokines and reactive intermediates. These elements are known to contribute to the advancement of DILI,^[^ ^21^ ^]^ highlighting the critical role of HERC2 in mitigating the condition's severity. Oxidative stress is characterized as a pathophysiological state, resulting from a discrepancy between the production of ROS and the inherent ability of the biological system to neutralize these reactive compounds. This imbalance sets off a cascade of detrimental cellular events, including mitochondrial dysfunction, the triggering of pro‐inflammatory signaling pathways, and ultimately leading to the induction of hepatocellular apoptosis or necrosis.^[^ ^5^, ^17^ ^]^ Indeed, the ablation of HERC2 in hepatocytes facilitates the APAP metabolism in vivo and in vitro, indicating that HERC2 directly modulates drug cytotoxicity in hepatocytes. In our present study, hepatocytes from APAP‐treated HERC2*^∆Alb^* mice exhibited a markedly augmented oxidative response relative to their counterparts. These findings suggest that HERC2 plays a pivotal role in maintaining redox homeostasis throughout the evolution of drug‐induced hepatotoxicity.

CYP2E1 is the central link between oxidative stress, ROS production, and hepatotoxic injury.^[^ ^8a^ ^]^ This enzyme can catalyze the metabolism of APAP to the hepatotoxic reactive metabolite, NAPQI, which results in liver injury.^[^ ^4b^ ^]^ Genetic variations in the CYP2E1 gene may influence an individual's susceptibility to DILI. It has been reported that CYP2E1‐null mice are highly resistant to APAP hepatoxicity.^[^ ^22^ ^]^ Studies with healthy human volunteers pre‐treated with disulfiram, a CYP2E1 inhibitor, further validate the role of CYP2E1 in the oxidation of APAP.^[^ ^4b^ ^]^ Consistent with these studies, our data showed elevated CYP2E1 levels in hepatocytes from APAP‐treated HERC2*^∆Alb^* mice, and its increased expression is associated with aggravated liver injury. A multitude of transcription factors and pro‐inflammatory cytokines have been identified as regulatory agents governing the expression of CYP2E1 in both hepatic and extrahepatic tissues.^[^ ^23^ ^]^ β‐catenin belongs to the armadillo family of proteins and is a critical component of the Wnt signaling pathway. The C‐terminal domain of β‐catenin contains the transactivation domain, which allows β‐catenin to act as a transcriptional regulator.^[^ ^24^ ^]^ We provided biochemical evidence that HERC2 directly interacted with β‐catenin. Indeed, β‐catenin influences the transcription of the murine CYP2E1 gene by activating the Wnt signaling pathway.^[^ ^7^ ^]^ The CYP2E1 Wnt‐responsive element is a DNA sequence within the promoter region of the CYP2E1 gene that is responsive to the activation of the Wnt signaling pathway.^[^ ^25^ ^]^ This element consists of a specific binding site for TCF/LEF transcription factors, which interact with β‐catenin to regulate gene expression.^[^ ^26^ ^]^ The association of β‐catenin with CYP2E1 Wnt‐responsive element within culminates in the subsequent recruitment of transcriptional co‐activators, thereafter facilitating the initiation of transcriptional activity and consequently augmenting the expression of CYP2E1.^[^ ^25^ ^]^ We, thus, assumed that HERC2‐mediated  reduced CYP2E1 production upon APAP challenge relies on the β‐catenin activity since inhibition of β‐catenin activity diminished the effect of HERC2 on APAP‐induced hepatotoxicity. A complex network of proteins tightly regulates the activation of β‐catenin. In the absence of Wnt signaling, β‐catenin is phosphorylated by a destruction complex comprising of Axin, adenomatous polyposis coli (APC), GSK‐3β, and casein kinase 1α (CK1α).^[^ ^27^ ^]^ This phosphorylation designates β‐catenin for ubiquitination, which precedes its targeted degradation via the proteasome pathway.^[^ ^27b^ ^]^ In the present study, our data showed HERC2 did not affect the protein level of GSK3β or the binding of GSK3β and β‐catenin, and the phosphorylation status of β‐catenin remains analogous irrespective of the presence or absence of HERC2. Alternatively, HERC2 functions as an E3 ubiquitin‐protein ligase, which accepts ubiquitin from an E2 ubiquitin‐conjugating enzyme as a thioester and subsequently directly transfers the ubiquitin to targeted substrates.^[^ ^14^, ^28^ ^]^ Wu et al. showed that HERC2 co‐localizes with and ubiquitinates the tumor suppressor BRCA1 in relation to the cell cycle and DNA damage response.^[^ ^14^ ^]^ Saez. et al. found that HERC2 is one of the most elevated E3 ubiquitin ligases in human embryonic stem cells (hESCs) compared to their differentiated counterparts.^[^ ^28^ ^]^ HERC2 interacts with LRRK2 to boost the recycling of the Notch ligand Delta‐like 1, consequently accelerating neural stem cell differentiation.^[^ ^29^ ^]^ McMillan et al. determined that HERC2 promotes the ubiquitination of Notch ligands through its Mind bomb (Mib) domain.^[^ ^30^ ^]^ Typically, K48‐linked polyubiquitination serves as a molecular signal that facilitates the targeted proteolysis of substrate proteins via the ubiquitin‐proteasome system. This post‐translational modification is pivotal for maintaining cellular homeostasis by directing aberrant or superfluous proteins for degradation.^[^ ^31^ ^]^ Our findings demonstrated that HERC2 augmented ubiquitination at the K48 residue of β‐catenin, which implied a role for HERC2 in propagating the proteasomal elimination of ubiquitin‐conjugated β‐catenin. Further interrogation into the functional interplay between HERC2 ubiquitin ligase activity and β‐catenin stability is warranted.

LNP is an effective delivery system to enable gene therapy and mRNA vaccine development in many preclinical and clinical studies.^[^ ^32^ ^]^ LNP achieves hepatocyte‐targeted delivery through apolipoprotein E (APOE)‐mediated binding and ensuing endocytic internalization by hepatocytes.^[^ ^33^ ^]^ This targeted delivery mechanism exploits the natural propensity of hepatocytes to uptake lipoprotein particles, thereby enhancing the specificity and efficacy of therapeutic cargo encapsulated within LNPs. Consequently, LNPs have been employed in therapeutic strategies targeting hepatic pathologies, including hepatocellular carcinoma and hepatic fibrosis.^[^ ^34^ ^]^ However, there is a paucity of literature addressing the utilization of LNPs in the context of acute hepatic injury, indicating a potential area for further research and application development. Herein, the data showed that LNP‐HERC2 was successfully delivered to hepatocytes, especially near the vein area, in which the hepatocytes were dramatically reduced in APAP acute liver failure patients. We postulated that LNP‐HERC2 preferentially accumulates in the vicinity of the central vein or portal vein areas, thereby reinforcing APAP resistance of hepatocytes and consequently conferring protection against APAP‐induced hepatic failure. DILI represents a primary causal factor underlying the need for hepatic transplantation.^[^ ^35^ ^]^ Prompt therapeutic intervention in the initial phases of drug‐induced toxicity holds the promise of preventing the progression to hepatic failure. Although significant strides have been made in understanding the molecular mechanisms driving the pathogenesis of DILI, developing agonists or antagonists that combine high specificity for their target molecules, effective absorption by hepatocytes, and a minimal profile of side effects continues to represent a substantial challenge in the field. Our findings have offered a strategic framework wherein LNP‐mediated therapy can confer targeted protection to hepatocytes from the hepatotoxic effects of drug overdose. Additional interrogation at the confluence of nanomedicine and gene therapy is necessitated to ascertain the prospects of LNPs as a versatile solution for the clinical management of DILI.

In HCC, hepatocytes undergo malignant transformation due to chronic conditions, while in DILI, hepatocytes suffer acute or subacute injury due to drug toxicity or immune responses. The roles and damage of hepatocytes in HCC and DILI differ significantly in their mechanisms, progression, and outcomes. The influence of certain molecules within hepatocytes on the progression of DILI and HCC can potentially be quite distinct. Hepatocytes specific knockout of Gab1, a kind of adaptor protein, led to increased mortality in APAP‐treated mice, which involved in limited AKT signal activation.^[^ ^36^ ^]^ Nevertheless, Gab1 was identified as one of the candidate cancer genes in driving epithelial‐mesenchymal transition of HCC^[^ ^37^ ^]^ and ablation of Gab1 expression in vitro restricted malignancy phenotype of HCC cells.^[^ ^38^ ^]^ Additionally, STAT3 is notable transcription factor that has demonstrated protective effects in various types of acute liver injury, such as toxin‐induced liver injury, LPS‐induced liver injury, and APAP‐induced DILI.^[^ ^39^ ^]^ Conversely, STAT3 also promotes tumor development,^[^ ^40^ ^]^ suggesting that caner‐promoting agent might present a potential therapeutic opportunity for DILI. In our study, we found that although HERC2 promoted tumorigenesis under chronic inflammation condition, it still served as a therapeutic target in DILI. By means of targeting delivery via LNPs, transient expression of HERC2 in hepatocytes helped these cells resist APAP‐induced hepatotoxicity. We will further investigate the therapeutic efficacy and safety of LNP‐based therapy in liver diseases in future studies.

In conclusion, our findings have illuminated that HERC2 seemed to play a pivotal role in drug metabolism by promoting β‐catenin degradation, which subsequently influenced the expression of CYP2E1. The data spotlight HERC2 as a prospective therapeutic target in DILI. Moreover, lipid nanoparticle‐enabled delivery of the HERC2 plasmid represents an exciting therapeutic avenue to alleviate DILI, showing its potential for clinical application in addressing this challenging condition.

## 4 Experimental Section

### Bioinformatics assay

The RDS file of human single‐nuclei RNA sequencing (snRNA‐seq) data was obtained from GSE223581 dataset. This dataset includes hepatocyte profiles from patients with APAP‐induced acute liver failure and healthy controls. Hepatocytes were clustered based on the previous study.^[^ ^41^ ^]^ HERC2 expression was displayed by density plot. Mouse single‐cell RNA sequencing data were sourced from dataset GSE200771, which comprises single‐cell sequencing data of mouse hepatocytes taken 2 hours after APAP injection. Hepatocytes were isolated from APAP‐treated or control mice for single‐cell sequencing. Seurat object was created by Seurat R package (version 4.0). The cells contained unique features between 200 to 5000 or under 25% mitochondrial counts were analyzed. HERC2 expression >0 was identified as the HERC2 positive group. Genome‐wide CRISPR‐Cas9 screen data were acquired from the GSE112463 dataset. BioGRID is the database of protein interaction (https://thebiogrid.org/). GeneRIF is the database of gene biological term distilled from biomedical publications (https://maayanlab.cloud/Harmonizome/dataset/GeneRIF+Biological+Term+Annotations).

### Animals

C57BL/6J mice were purchased from the Laboratory Animal Center of Southern Medical University (Guangzhou, China). Hepatocyte‐specific HERC2 knockout mice were established in the previous work.^[^ ^12a^ ^]^ All mice were housed at a constant temperature (19‐23 °C) and 55 ± 10% humidity under a 12 hours light/dark cycle with free access to water and commercial feed. Animal experiments were approved by the Welfare and Ethical Committee for Experimental Animal Care of Southern Medical University (2020066). Mice were randomly divided into each group to perform animal experiments.

Eight‐week‐old male mice were fasted overnight and intraperitoneally injected with APAP (sc‐203425A, Santa Cruz Biotechnology, Santa Cruz, CA, USA) at the dose of 400 mg kg^−1^ to induce hepatotoxicity^[^ ^42^ ^]^ and at the dose of 700 mg kg^−1^ to evaluate survival rate.^[^ ^43^ ^]^ APAP was dissolved in phosphate‐buffered saline (PBS) and the vehicle mice was treated with equal volume of PBS as APAP. For LNPs delivery, LNPs (200µl) were mixed with plasmids (20 µg). The LNP‐DNA complex (200µl) was injected into mice via tail vein 2 hours before APAP administration.

The serum was collected for serum glutamic pyruvic transaminase (SGPT) (C009‐2‐1, Jiancheng Biotech, Nanjing, China) and serum glutamic oxaloacetic transaminase (SGOT) (C010‐2‐1, Jiancheng Biotech) assay. The tissue homogenates from mice livers were used to evaluate the levels of MDA with commercial kits (A003‐1‐2, Jiancheng Biotech). Liver damage was detected by hematoxylin and eosin (H&E) staining.

### Cell Culture and Treatments

HepaRG cell line was a reliable human cell line to study mechanisms of APAP hepatotoxicity and was obtained from Thermo Fisher Scientific, which is a trademark of BioPredic International. The cells have been terminally differentiated and provided as the company claimed. HEK293T cell line was obtained from the ATCC. All the cell lines have been confirmed authentic by short tandem repeat (STR) profiling and tested for mycoplasma negativity by PCR analysis (C0301S, Beyotime Biotechnology, Shanghai, China). Cells were cultured in DMEM (11965092, Thermo Fisher Scientific, Carlsbad, CA, USA) supplemented with 10% fetal bovine serum (FBS), penicillin (100 U/ml), and streptomycin (100 µg ml^−1^), at 37 °C in a humidified atmosphere containing 5% CO_2_. Human HERC2 knockout (Cas9) (sc‐405015) or overexpressed (SAM) (sc‐405015‐ACT) Crispr plasmids and mouse HERC2 overexpressed (SAM) (sc‐420831‐ACT) Crispr plasmids were purchased from Santa Cruz. Poly‐clonal cells were used in the experiments. The HA‐Ub, HA‐Ub‐K48, HA‐Ub‐K63, HA‐Ub‐K33, and mouse GFP labeled vector plasmids were purchased from Addgene (http://www.addgene.org). The HA‐HERC2, FLAG‐β‐catenin, MYC‐HERC2 plasmids were constructed by ourselves by means of the Snapgene software. The sequence of the plasmids was provided in the supplementary files. The plasmids were transiently transfected by the lipofectamine 3000 (L3000015, Thermo Fisher Scientifc, Inc.) followed by the instructions.

### High‐Performance Liquid Chromatography (HPLC) Analysis

Liver tissues were collected to detect APAP‐adducts level by high‐performance liquid chromatography (HPLC; Agilent1260, CA, USA), as described in a previous study.^[^ ^44^ ^]^ Briefly, samples were treated with methanol, followed by centrifuging at 16 000 g for 15 minutes. The supernatant was vacuum‐dried and reconstituted in methanol. A total volume of 10µl sample was injected into a Hypersil ODS (C18) column (Thermo Scientific). To detect APAP‐adducts, the mobile phase was composed of methanol and water at a volume of 50:50. The column was kept a flow rate of 1.0 mL min^−1^ at 37 °C. Data were analyzed using Agilent LC1260 software.

### Flow Cytometry

To measure the mitochondria and intracellular oxidative stress level, hepatocytes isolated from mice liver or HepaRG cells were incubated with 1µm Mitosox indicator (M36009, Thermo Fisher Scientific) or 10µM DCFH‐DA indicator (S0033S, Beyotime Biotechnology) in the dark for 30 minutes at 37 °C. The cells were acquired and analyzed using the BD FACSDiva program in the flow cytometry FACS LSRFortessa (BD Biosciences, San Jose, CA, USA). Hepatocytes were isolated as described previously.^[^ ^45^ ^]^ Briefly, mouse livers were initially perfused in situ via the portal vein with a calcium‐free buffer solution, followed by perfusion with 0.05% type IV collagenase. Subsequently, the livers were excised and gently dissociated. The resulting cell suspension was filtered through a 70‐µm polyamide mesh to remove tissue debris. Hepatocytes were then purified by three consecutive low‐speed centrifugation steps (50 × g for 2 minutes each), with careful removal of the supernatant after each centrifugation.

### RT‐qPCR Analysis

The total RNA of cells or liver tissues was extracted in 1 ml TRIzol (Thermo Fisher Scientific) based on the manufacturer's instructions. Then, cDNA was synthesized at 50 °C for 10 minutes and 85 °C for 5 seconds. SYBR Green (A46112, Thermo Fisher Scientific) was applied for qPCR according to the following conditions: initial denaturation at 94 °C for 30 seconds, followed by 35 cycles of denaturation at 94 °C for 5 seconds, and extension at 60 °C for 30 seconds. The expression of the target genes was normalized to β‐actin and determined by the 2^−∆∆Ct^ method. All the sequences of primers involved in this study were provided as Table S1 (Supporting Information).

### Immunoprecipitation and Western Blot

Whole‐cell lysates were extracted by cell lysis buffer (P0013, Beyotime Biotechnology) and then quantified by BCA assay (23225, Thermo Fisher Scientific). For the immunoprecipitation assay, protein was incubated with 1 µg antibody at 4 °C overnight, and incubated with protein A/G agarose (Santa Cruz Biotechnology) for another 2 hours at 4 °C. The eluted precipitants were tested by SDS‐PAGE.

For western blot analysis, samples were separated by SDS‐PAGE and transferred onto PVDF membranes. The membranes were blocked by 5% BSA for 1 hour at room temperature, then incubated with the indicated primary antibodies at 4 °C overnight. Subsequently, the membranes were incubated with HRP‐conjugated secondary antibody at room temperature for 1 hour. Antibodies used in this study were as follows: HERC2 (sc‐515891, Santa Cruz Biotechnology), CYP2E1 (19937‐1‐AP, Proteintech, Wuhan, China), APAP‐adducts (OAEF00896, Aviva Systems Biology Corporation, San Diego, CA, USA), β‐catenin (51067‐2‐AP, Proteintech), GSK3β (22104‐I‐AP, Proteintech), p‐β‐catenin (28776‐1‐AP, Proteintech), FLAG (66008‐4‐Ig, Proteintech), HA (66006‐2‐Ig, Proteintech), MYC (16286‐1‐AP, Proteintech), Ub (sc‐8017, Santa Cruz), β‐actin (66009‐1‐Ig, Proteintech).

### CCK‐8 Assay

Cell viability was detected by Cell Counting Kit‐8 (CCK‐8, Dojindo Molecular Technologies, Japan) based on the manufacturer's protocols. Briefly, cells were planted into 96‐well plates, and incubated with CCK8 solution (10µl) at 37 °C for 2 hours before the end of the experiment. The 450 nm absorbance was detected.

### Immunohistochemistry

Slides were hydrated, and an antigen retrieval procedure was performed in citrate buffer (pH 6.0) at 100 °C for 10 minutes. To block endogenous peroxidase activity, slides were incubated with 3% H_2_O_2_ at room temperature for 15 minutes, then blocked with goat serum at 37 °C for 1 hour and incubated with the indicated antibody at 4 °C overnight. Then, the slides were stained with HRP‐conjugated secondary antibody at 37 °C for 1 hour. An enhanced diaminobenzidine kit (GK500710, Gene Tech, Shanghai, China) was used to determine the immunoreactivity, and the nuclear was stained with hematoxylin.

### Immunofluorescence

The cells were fixed and permeabilized, followed by blocking with goat serum for 1 hour. Then, the cells were stained with primary antibodies overnight at 4 °C. Subsequently, the cells were incubated with goat anti‐mouse IgG H&L (Alexa Fluor 488) (ab150113, Abcam, Waltham, MA, USA) or goat anti‐rabbit IgG H&L (Alexa Fluor 647) (ab150079, Abcam) antibodies for 1 hour at 37 °C. The nucleus was stained with DAPI for 30 minutes at 37 °C. The images were obtained with a 63× oil immersion objective on a LSM900 Carl Zeiss Microscope.

### Preparation of LNPs

LNPs were constructed by the solvent diffusion method as previously described. In brief, 3 mg DOTAP, 0.75 mg PEG‐distearoyl phosphatidylethanolamine (DSPE), 10.5 mg dipalmitoylphosphatidylcholine (DPPC), and 0.75 mg cholesterol were dissolved in 1.5 ml ethanol and incubated at 60 °C. Subsequently, the solution was rapidly dispersed in 15 mL, 0.1% w/v F68 solution at 60 °C in the condition of 400 rpm stirring for 5 minutes. To form the LNPs/plasmids complex, the solution was mixed with plasmids and placed at room temperature for 30 minutes. The size distribution and zeta potential of the complex were determined by dynamic light scattering (DLS) spectrometer (3000HS; Malvern Instruments, Malvern, UK).

### Statistical Analysis

GraphPad Prism 8.0.1 was used for statistical analyses. Kaplan–Meier and log‐rank tests were applied to determine survival rates. The data are displayed as the mean ± standard deviation (SD). Two tailed‐unpaired Student's t test was performed to compare the difference between two unpaired groups. *p* < 0.05 was considered to indicate a statistically significant difference. All experiments were independently repeated in triplicate. Sample size of each group was indicated in the figure legends.

## Conflict of Interest

The authors declare no conflict of interest.

## Author Contributions

Y.L., Q.X., and Y.L. contributed equally to this manuscript. DZ, QC, and YT designed experiments. Y.L., Q.X., Y.L., S.C., Z.Z., J.L., M.L., and L.Z. performed the experiments. D.Z., Q.C., Y.L., J.Z., Y.T., X.L., and Y.T. analyzed the data. X.L., L.Z., and Y.T. provided technical support. D.Z., Y.L., Q.C., and Y.T. wrote the paper. All authors reviewed and approved the manuscript prior to submission.

## Supporting information



Supporting Information

## Data Availability

The data that support the findings of this study are available from the corresponding author upon reasonable request.
