# Supplementary material for: Hepatocyte‐Targeted Lipid Nanoparticle Delivery of HERC2 Plasmid Controls Drug‐Induced Hepatotoxicity by Limiting β‐Catenin‐Regulated CYP2E1 Expression
Source: Adv Sci (Weinh). 2024 Oct 23;11(46):2401633. doi: 10.1002/advs.202401633 (PMC11633468; doi:10.1002/advs.202401633)
Supplement: Supplementary file 1 — Supporting Information [file ADVS-11-2401633-s001.docx]

**Supplemental information**

**Hepatocyte-Targeted Lipid Nanoparticle Delivery of HERC2 Plasmid Controls Drug-Induced Hepatotoxicity by Limiting β-catenin-Regulated CYP2E1 Expression**

Yunzhi Liu, Qishan Xu, Yan Liu, Sihang Cao, Zhuojun Zheng, Jialiang Luo, Jia Zhou, Xiao Lu, Liyun Zhang, Yanan Tan, Qingyun Chen, and Daming Zuo

**Supplemental Figure S1**


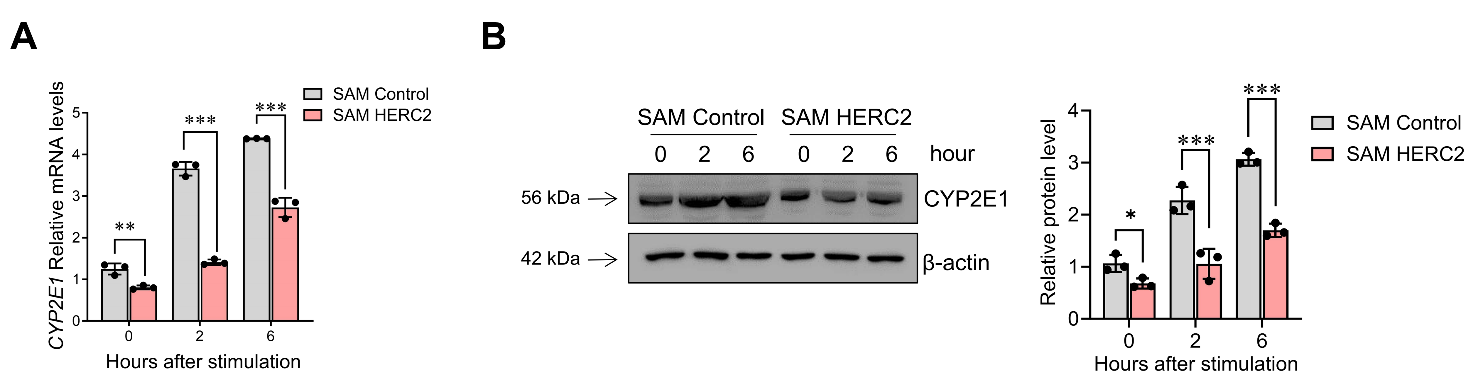


**Fig. S1 HERC2 restricted CYP2E1 expression in hepatocytes upon APAP stimulation *in vitro*.**

HERC2-overexpressed HepaRG cells and control cells were treated with APAP at the dose of 20 mM. **(A)** The mRNA level of CYP2E1 was detected by quantitative RT-PCR analysis. **(B)** The protein level of CYP2E1 was evaluated by western blotting. **p*<0.05, ***p*<0.01, ****p*<0.001. Data from one representative experiment of three independent experiments are presented. The data are displayed as the mean ± standard deviation (SD). Unpaired Student’s t test was used.

**Supplemental Figure S2**

**
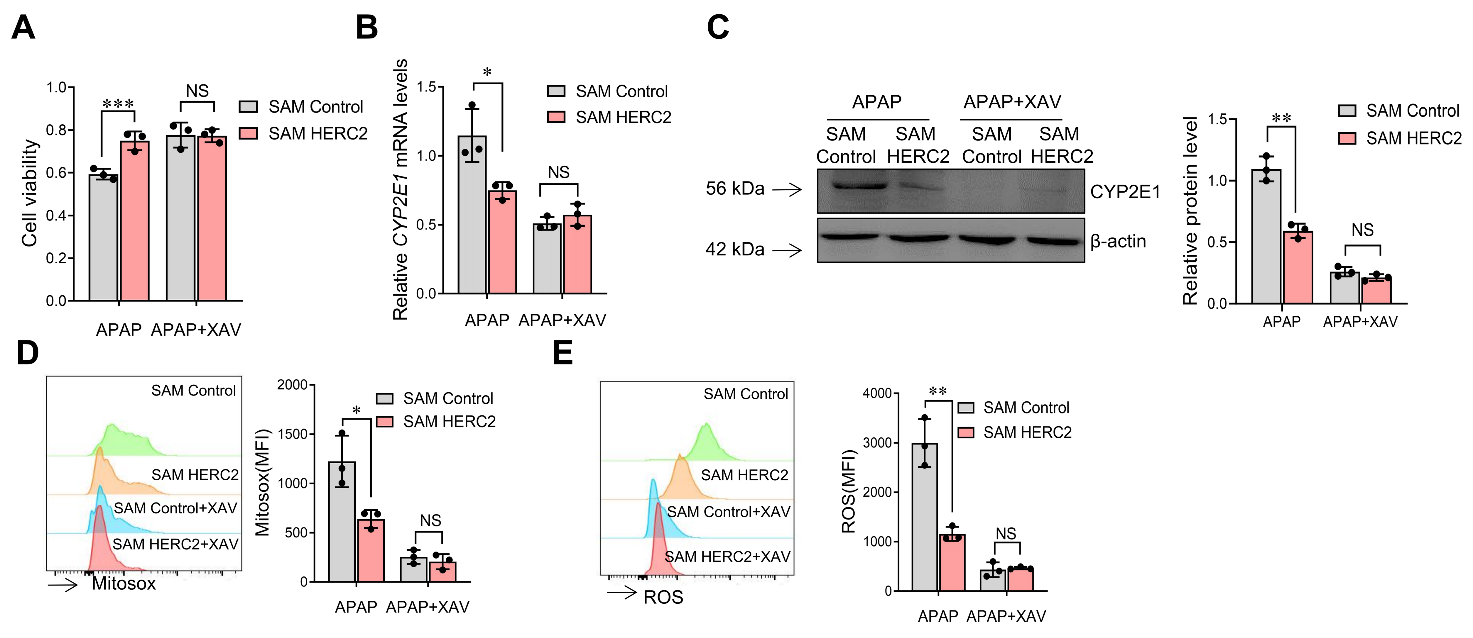
**

**Fig. S2 HERC2 regulated APAP hepatoxicity through β-catenin signaling.**

HERC2-overexpressed HepaRG cells and control cells were treated with 2 μM XAV939 for 4 hours before 20 mM APAP treatment. **(A)** The cell viability was determined by the CCK-8 assay. **(B)** The mRNA level of CYP2E1 was detected by quantitative RT-PCR analysis. **(C)** The protein level of CYP2E1 was evaluated by western blotting. **(D, E)** The mitochondrial ROS level **(D)** and intercellular ROS level **(E)** in hepatocytes were determined by flow cytometry analysis. NS: not significant, **p*<0.05, ***p*<0.01, ****p*<0.001. Data from one representative experiment of three independent experiments are presented. The data are displayed as the mean ± standard deviation (SD). Unpaired Student’s t test was used.

**Supplemental Figure S3**


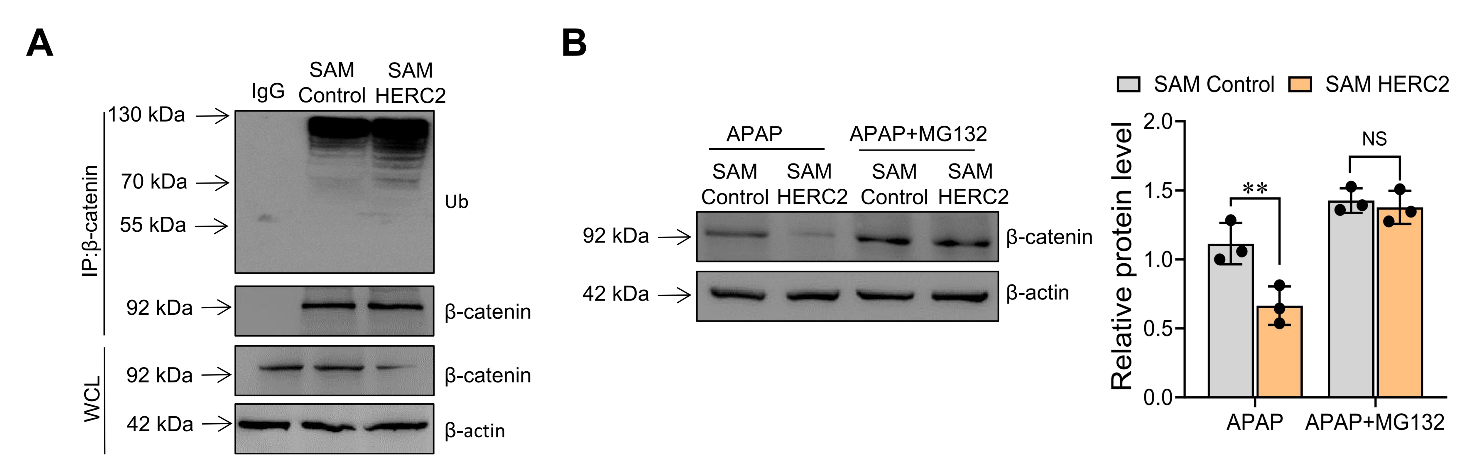


**Fig. S3 HERC2 mediated β-catenin ubiquitination.**

HERC2-overexpressed (SAM HERC2) HepaRG cells and control cells were treated with 20 mM APAP and 10 μmol MG132 for 6 hours. **(A)** The ubiquitination level of β-catenin was determined by immunoprecipitation. **(B)** The protein level of β-catenin was evaluated by western blotting. NS: not significant, ***p*<0.01. Data from one representative experiment of three independent experiments are presented.

**Supplemental Figure S4**


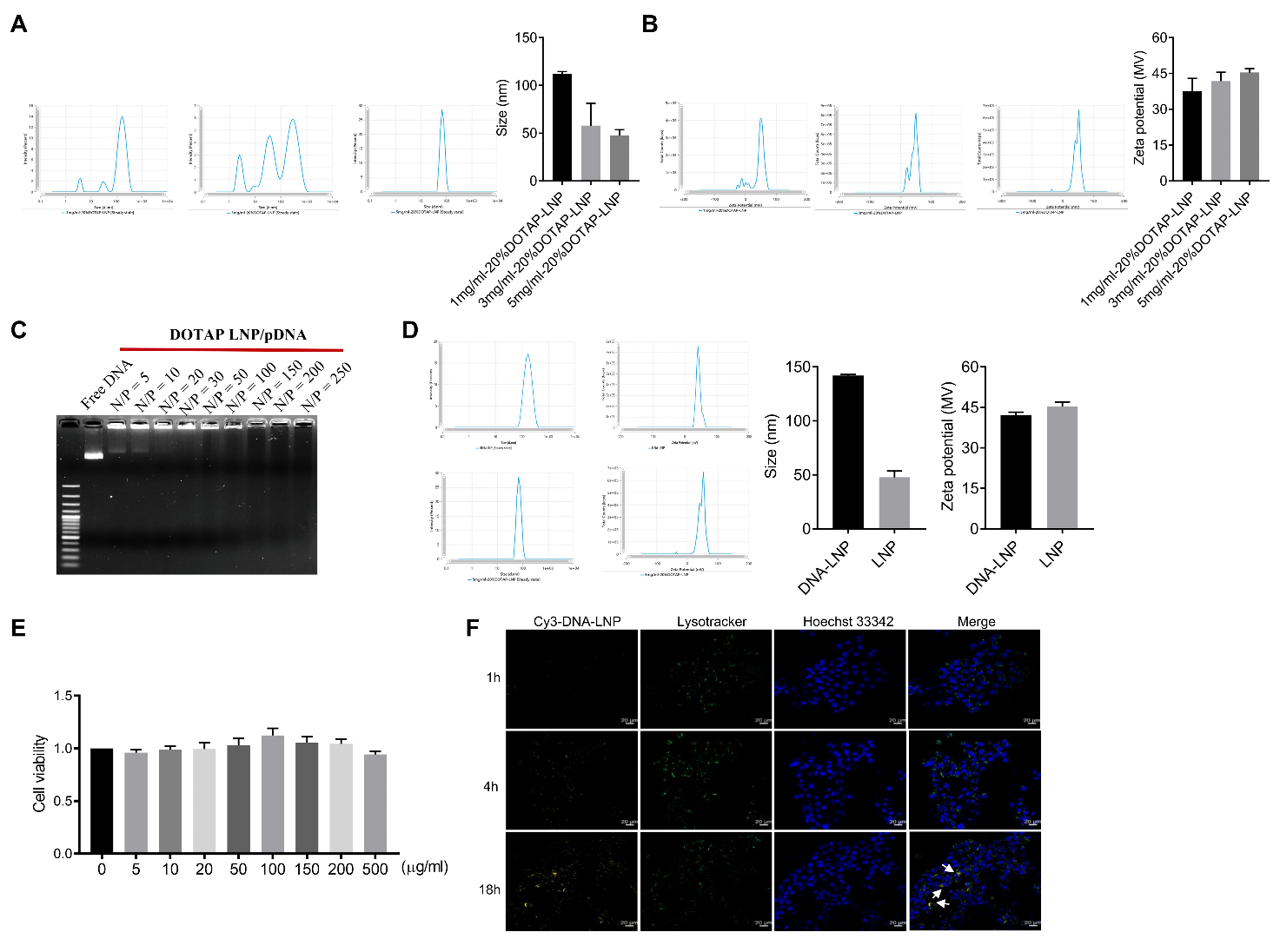


**Fig. S4 Targeted delivery of LNPs into hepatocytes.**

**(A)** The particle size of different concentrations of 20% DOTAP-LNP. **(B)** Zeta potential of different concentrations of 20% DOTAP-LNP. **(C)** Agarose gel electrophoresis analyzed the stability of the DOTAP-LNP-DNA complex. **(D)** Particle size and zeta potential of DOTAP-LNP-DNA complex. **(E)** LNPs were cultured with HepaRG cells for 24 hours, and CCK-8 assay was performed to detect cell viability. **(F)** LNPs were cultured with HepaRG cells for 18 hours, lysotracker was used to analyze the lysosome escape ability of LNPs. Data from one representative experiment of three independent experiments are presented. The data are displayed as the mean ± standard deviation (SD).

**Supplementary Table**

**Tab.S1 Primers for the target genes.**

| Gene | Forward primer (5’🡪3’) | Reverse primer (5’🡪3’) |
| --- | --- | --- |
| (m)Il-6 | TAGTCCTTCCTACCCCAATTTCC | TTGGTCCTTAGCCACTCCTTC |
| (m)Tnf-α | CCCTCACACTCAGATCATCTTCT | GCTACGACGTGGGCTACAG |
| (m)CYP2E1 | CGTTGCCTTGCTTGTCTGGA | AAGAAAGGAATTGGGAAAGGTCC |
| (h)CYP2E1 | ATGTCTGCCCTCGGAGTCA | CGATGATGGGAAGCGGGAAA |
| (m) β-catenin | ATGGAGCCGGACAGAAAAGC | TGGGAGGTGTCAACATCTTCTT |
| (h) β-catenin | AAAGCGGCTGTTAGTCACTGG | CGAGTCATTGCATACTGTCCAT |
| (m) β-actin | GGCTGTATTCCCCTCCATCG | CCAGTTGGTAACAATGCCATGT |
| (h) β-actin | CATGTACGTTGCTATCCAGGC | CTCCTTAATGTCACGCACGAT |
